# Polydopamine nanoparticles as immunomodulators: inhibition of M1 microglial polarization

**DOI:** 10.3389/fbioe.2025.1672520

**Published:** 2025-10-17

**Authors:** Maria Cristina Ceccarelli, Luigi Lai, Alessio Carmignani, Matteo Battaglini, Gianni Ciofani

**Affiliations:** ^1^ Istituto Italiano di Tecnologia, Smart Bio-Interfaces, Pontedera, Italy; ^2^ Scuola Superiore Sant’Anna, The Biorobotics Institute, Pontedera, Italy; ^3^ Politecnico di Torino, Department of Mechanical and Aerospace Engineering, Torino, Italy

**Keywords:** polydopamine nanoparticles, microglia, immunomodulation, neuroinflammation, antioxidant

## Abstract

Neuroinflammation is a central feature of numerous neurodegenerative diseases, including Alzheimer’s and Parkinson’s disease, where excessive activation of microglia can contribute to neuronal damage. The pro-inflammatory M1 phenotype of microglia is characterized by increased production of reactive oxygen species (ROS), overexpression of surface markers such as CD40 and CD86, and secretion of cytokines like IL-6, IL-8, and TNF-α, all of which exacerbate oxidative stress and neurodegeneration. The development of strategies to control and tune microglial pro-inflammatory activation is therefore critical for reducing the progression of these conditions. In this study, the potential of polydopamine nanoparticles (PDNPs) as novel immunomodulatory agents for attenuating M1 microglial polarization was investigated. PDNPs were synthesized via a simple and reproducible protocol and thoroughly characterized in terms of size, morphology, hydrodynamic diameter, and surface charge, confirming their uniformity and stability. Biocompatibility assays showed that PDNPs are well tolerated by human microglial clone 3 (HMC3) cells, with minimal cytotoxicity even at relatively high concentrations. Confocal microscopy and flow cytometry analyses demonstrated efficient internalization of PDNPs by microglia, with preferential accumulation in lysosomal compartments and negligible mitochondrial localization. To mimic neuroinflammatory conditions, HMC3 cells were stimulated with interferon-gamma (IFN-γ), which significantly increased intracellular ROS levels, surface expression of CD40 and CD86, and secretion of pro-inflammatory cytokines. The co-treatment with PDNPs effectively mitigated these effects by reducing oxidative stress, suppressing the upregulation of M1 markers, and decreasing cytokine release, thereby preventing the shift toward a pro-inflammatory state. The results of this work demonstrate that PDNPs not only exhibit excellent biocompatibility and cellular uptake but also provide a robust means of counteracting IFN-induced microglial activation. These results establish PDNPs as promising nanoplatforms for modulating neuroinflammation and microglial activation. This study highlights the potential of PDNPs for future applications in the treatment of neurodegenerative diseases.

## 1 Introduction

Neurodegenerative diseases such as Alzheimer’s disease (AD), Parkinson’s disease (PD), and amyotrophic lateral sclerosis (ALS) are progressive, incurable disorders marked by the loss of neuronal function and structure. (Dickson, 2018; Hansen et al., 2018; Rowland and Shneider, 2001). Cognitive and motor impairments characterize these conditions and represent a growing burden for healthcare systems worldwide, particularly in aging populations. (Ding et al., 2022). Despite the molecular and cellular differences present among these disorders, they share similar pathological mechanisms, including chronic neuroinflammation and elevated oxidative stress, both of which contribute to neuronal dysfunction and degeneration. (Martinelli et al., 2020). Microglia, the resident immune cells of the central nervous system (CNS), are one of the primary types of cells involved in neuroinflammatory responses. (Kettenmann et al., 2011). Microglia maintains homeostasis in the healthy brain, continuously controlling their environment and responding to injury or infection. Upon activation, microglia undergo phenotypic polarization with two main states: the pro-inflammatory M1 phenotype and the anti-inflammatory M2 phenotype. (Guo et al., 2022). While transient M1 activation plays a beneficial role in clearing pathogens and cellular debris, sustained M1 polarization leads to the release of neurotoxic moieties, including reactive oxygen species (ROS), tumor necrosis factor-α (TNF-α), interleukin-6 (IL-6), and interleukin-8 (IL-8). (Guo et al., 2022; Tang and Le, 2016; Honjoh et al., 2019; Ransohoff, 2016). These factors can increase oxidative stress, disrupt synaptic function, and promote further neuronal injury, establishing a self-amplifying cycle of inflammation and degeneration. (Guo et al., 2022; Tang and Le, 2016; Honjoh et al., 2019; Ransohoff, 2016). The ability to modulate microglial activation, specifically to suppress or prevent the M1 phenotype, is therefore a promising therapeutic strategy for mitigating neuroinflammation and slowing disease progression. (Battaglini et al., 2024a). In recent years, interest has grown in the use of nanotechnology to target immune responses within the CNS. Among emerging nanomaterials with therapeutic potential, polydopamine nanoparticles (PDNPs) have attracted increasing attention due to their unique combination of biocompatibility, antioxidant activity, and versatility. (Battaglini et al., 2024b). Polydopamine (PDA) is a synthetic polymer inspired by the adhesive proteins of mussels, formed through the oxidative self-polymerization of dopamine under mildly alkaline conditions. (Lee et al., 2007; Liu et al., 2013). Structurally similar to melanin, PDA contains abundant catechol, quinone, and amine functional groups, which not only allow strong adhesion to a wide range of surfaces but also confer potent free radical scavenging capacity. (Guo et al., 2021). When synthesized at the nanoscale, PDNPs present the main properties of bulk PDA but gain significant advantages in terms of surface area, dispersibility, and cellular uptake. PDNPs are, in fact, fully organic, biodegradable, and stable in aqueous environments. (Battaglini et al., 2024b). Moreover, their catechol and quinone groups act as electron donors and acceptors, enabling them to neutralize ROS and reduce oxidative stress within cells. (Battaglini et al., 2024b; Carmignani et al., 2024; Battaglini et al., 2020; Battaglini et al., 2022; Carmignani et al., 2025). Importantly, unlike many inorganic nanomaterials, PDNPs do not rely on metallic cores or surface coatings to achieve their effects, minimizing the risk of long-term toxicity. Beyond their antioxidant function, PDNPs can modulate cellular responses (Battaglini et al., 2020; Carmignani et al., 2025), yet their impact on immune cells of the central nervous system (particularly microglia) has not been systematically explored. This presents a significant research opportunity, as modulating the inflammatory activity of microglia using nanomaterials could offer a targeted and biocompatible approach to reducing neuroinflammation. In the present study, we examine whether PDNPs can prevent the M1 polarization of microglia induced by interferon-gamma (IFN-γ), a key inflammatory cytokine. By evaluating PDNP uptake, localization, and their impact on ROS production and inflammatory signaling in HMC3 cells, we aim to establish PDNPs as multifunctional nanotherapeutics capable of both protecting microglia from oxidative stress and modulating their immune phenotype in the context of neuroinflammation.

## 2 Materials and methods

### 2.1 Synthesis and characterization of PDNPs

PDNPs were synthesized via the oxidative self-polymerization of dopamine in an alkaline aqueous medium. (Bao et al., 2018). Briefly, 90 mL of Milli-Q water and 40 mL of ethanol were combined under gentle magnetic stirring. To this mixture, 10 mL of Milli-Q water containing 0.5 g of dopamine hydrochloride (Sigma-Aldrich) was added, followed by the addition of 2 mL of ammonium hydroxide solution to initiate the polymerization process. The reaction was maintained at room temperature under continuous stirring for 24 h to allow complete nanoparticle formation. Following synthesis, the suspension was diluted 1:4 with ethanol and subjected to centrifugation at 7960 *g* for 5 min at room temperature to pellet the nanoparticles. The pellet was subsequently washed three times with Milli-Q water by centrifugation at 21000 *g* for 30 min at 4 °C to remove residual reactants. Purified PDNPs were lyophilized, and their concentration was determined by weighing freeze-dried aliquots. The morphological characteristics of the PDNPs were examined using scanning electron microscopy (SEM). For sample preparation, 10 μL of PDNP suspension (100 μg/mL) was deposited onto a silicon substrate and allowed to air-dry under a chemical hood. The dried samples were gold-coated using a Quorum Tech Q150RES sputter coater operating at 30 mA for 60 s. SEM imaging was performed on a Helios NanoLab 600i dual-beam SEM/FIB system (FEI), and particle size analysis was conducted using Gwyddion software. Hydrodynamic diameter, polydispersity index (PDI), and ζ-potential of PDNPs were assessed by dynamic light scattering (DLS) using a Malvern Zetasizer Nano ZS90 (Malvern Instruments). All measurements were performed on freshly prepared PDNP suspensions at a concentration of 100 μg/mL in Milli-Q water. Disposable polystyrene cuvettes were employed for size and PDI determination, while folded capillary cells were used for ζ-potential analysis.

### 2.2 HMC3 cell culture

Human microglial clone 3 (HMC3) cells were maintained under standard proliferative conditions in high-glucose Minimum Essential Medium (MEM; Sigma-Aldrich) supplemented with 10% heat-inactivated fetal bovine serum (FBS; EuroClone), 1% L-glutamine (200 mM stock; Gibco), and 1% penicillin–streptomycin (100 IU/mL penicillin, 100 μg/mL streptomycin; Gibco). Cultures were incubated at 37 °C in a humidified atmosphere containing 5% CO_2_. The culture medium was refreshed every 2 days, and cells were passaged upon reaching 80%–90% confluency.

### 2.3 Cell viability and proliferation assays

The impact of PDNPs on HMC3 cell viability and proliferation was assessed using the Live/Dead Viability/Cytotoxicity Kit (Thermo Fisher) and the Quant-iT™ PicoGreen™ dsDNA Assay Kit (Invitrogen). For the Live/Dead assay, HMC3 cells were seeded at a density of 10000 cells/cm^2^ in 24-well plates (Corning) and allowed to adhere for 24 h. Cells were then exposed to PDNPs at concentrations of 0, 31.25, 62.5, 125, and 250 μg/mL for 72 h. Following treatment, cells were washed with Dulbecco’s phosphate-buffered saline (DPBS; EuroClone) and incubated for 30 min at 37 °C in phenol red-free medium containing Hoechst 33342 (5 μg/mL; Invitrogen), ethidium homodimer-1 (4 µM), and calcein-AM (2 μM; Thermo Fisher). After staining, cells were rinsed with DPBS and imaged using a Nikon Eclipse Ti fluorescence microscope equipped with a 10x objective. Live and dead cells were quantified using ImageJ software. For DNA quantification, the PicoGreen™ assay was performed. HMC3 cells were seeded at the same density (10000 cells/cm^2^) in 96-well plates (Sarstedt), allowed to adhere for 24 h, treated with PDNPs at the concentrations listed above, and incubated for 72 h. After treatment, cells were washed with DPBS, resuspended in 100 µL of Milli-Q water, and subjected to three freeze–thaw cycles (−80 °C–37 °C). The PicoGreen™ dsDNA assay was performed in black 96-well polystyrene plates (Corning Costar) following the manufacturer’s protocol. After 10 min of incubation at room temperature, fluorescence intensity was measured using a Victor X3 Multilabel Plate Reader (PerkinElmer).

### 2.4 Cellular internalization

The uptake and intracellular localization of PDNPs were evaluated using flow cytometry and confocal laser scanning microscopy following fluorescent labeling of the nanoparticles. For nanoparticle labeling, 20 µM DiO dye (Vybrant™ Multicolor Cell-Labeling Kit; Thermo Fisher) was added to 1 mL of Milli-Q water containing PDNPs at a concentration of 7 mg/mL. The suspension was stirred at room temperature for 3 h to allow dye incorporation. Excess dye was removed by washing the nanoparticles three times with Milli-Q water via centrifugation at 21300 *g* for 90 min at 4 °C. For flow cytometric analysis, HMC3 cells were seeded at a density of 15000 cells/cm^2^ in 24-well plates and allowed to adhere for 24 h. Cells were then incubated with phenol red-free medium containing DiO-labeled PDNPs (100 μg/mL) for different time intervals (4 h, 24 h, and 72 h). After incubation, cells were detached by trypsinization, washed with DPBS, and analyzed using a CytoFLEX flow cytometer (Beckman Coulter). Fluorescence intensity was compared with that of untreated controls to quantify the internalization of nanoparticles. For confocal microscopy, cells were seeded at the same density in WillCo glass-bottom dishes and allowed to adhere for 24 h. Following incubation with DiO-labeled PDNPs (100 μg/mL) for 4, 24, or 72 h, cells were rinsed with DPBS and stained with either Hoechst 33342 (5 μg/mL; Invitrogen) and LysoTracker Red (5 μM; Thermo Fisher) for lysosomal imaging, or Hoechst 33342 (5 μg/mL) and tetramethylrhodamine methyl ester (TMRM; 0.05 µM; Life Technologies) for mitochondrial visualization. Staining was performed at 37 °C for 30 min, followed by two washes with DPBS. Images were acquired using a Nikon C2s confocal microscope equipped with a ×60 oil immersion objective.

### 2.5 Cellular phenotype analysis

To evaluate the effect of PDNPs on microglial phenotype, HMC3 cells were seeded at a density of 10,000 cells/cm^2^ in 6-well plates and allowed to adhere for 24 h. Cells were then divided into four experimental groups: untreated control, PDNPs alone (100 μg/mL), IFN-γ alone (0.6 μg/mL), and PDNPs + IFN-γ (100 μg/mL and 0.6 μg/mL, respectively). After 72 houeùrs of incubation, cells were harvested, washed with DPBS, and stained with 10 μg/mL FITC-conjugated anti-CD40 antibody (Abcam) for 30 min at 4 °C. Following staining, cells were rinsed with DPBS and analyzed using a CytoFLEX flow cytometer (Beckman Coulter). The same procedure was applied for CD86 detection using a FITC-conjugated anti-CD86 antibody (Abcam).

### 2.6 Assessment of PDNP antioxidant properties

The antioxidant capacity of PDNPs was determined by measuring intracellular ROS. HMC3 cells were seeded under the same conditions as above, treated with PDNPs (100 μg/m), IFN-γ (0.6 μg/mL), or PDNPs + IFN-γ for 72 h, and stained with 2.5 μM CellROX™ Green Reagent (Invitrogen) for 30 min in phenol red-free medium. After staining, cells were detached, washed, and resuspended in DPBS. Fluorescence intensity was measured using a CytoFLEX flow cytometer and compared across conditions.

### 2.7 Pro-inflammatory cytokine quantification

The secretion of TNF-α, IL-6, and IL-8 was quantified using ELISA kits (Invitrogen) according to the manufacturer’s instructions. Briefly, HMC3 cells were seeded at 10,000 cells/cm^2^ in 6-well plates, allowed to adhere for 24 h, and treated as described above (PDNPs, IFN-γ, PDNPs + IFN-γ) for 72 h. After incubation, culture media were collected and centrifuged at 16,000 X g for 10 min to remove debris. Supernatants were used for ELISA, and absorbance was measured using a Victor X3 Multilabel Plate Reader (PerkinElmer) at λ_ex = 502 nm and λ_em = 523 nm. Cytokine concentrations were normalized to cell number, which was determined by manual counting using a Bürker chamber.

### 2.8 Statistical analysis

Statistical analyses were performed using R software. Data normality was assessed with the Shapiro–Wilk test. For data that followed a normal distribution, one-way ANOVA was applied, followed by an LSD *post hoc* test with Bonferroni correction for multiple comparisons. Results are reported as mean ± standard error. Unless otherwise stated, all experiments were carried out in triplicate (n = 3).

## 3 Results

### 3.1 Synthesis and characterization of PDNPs

**FIGURE 1:**
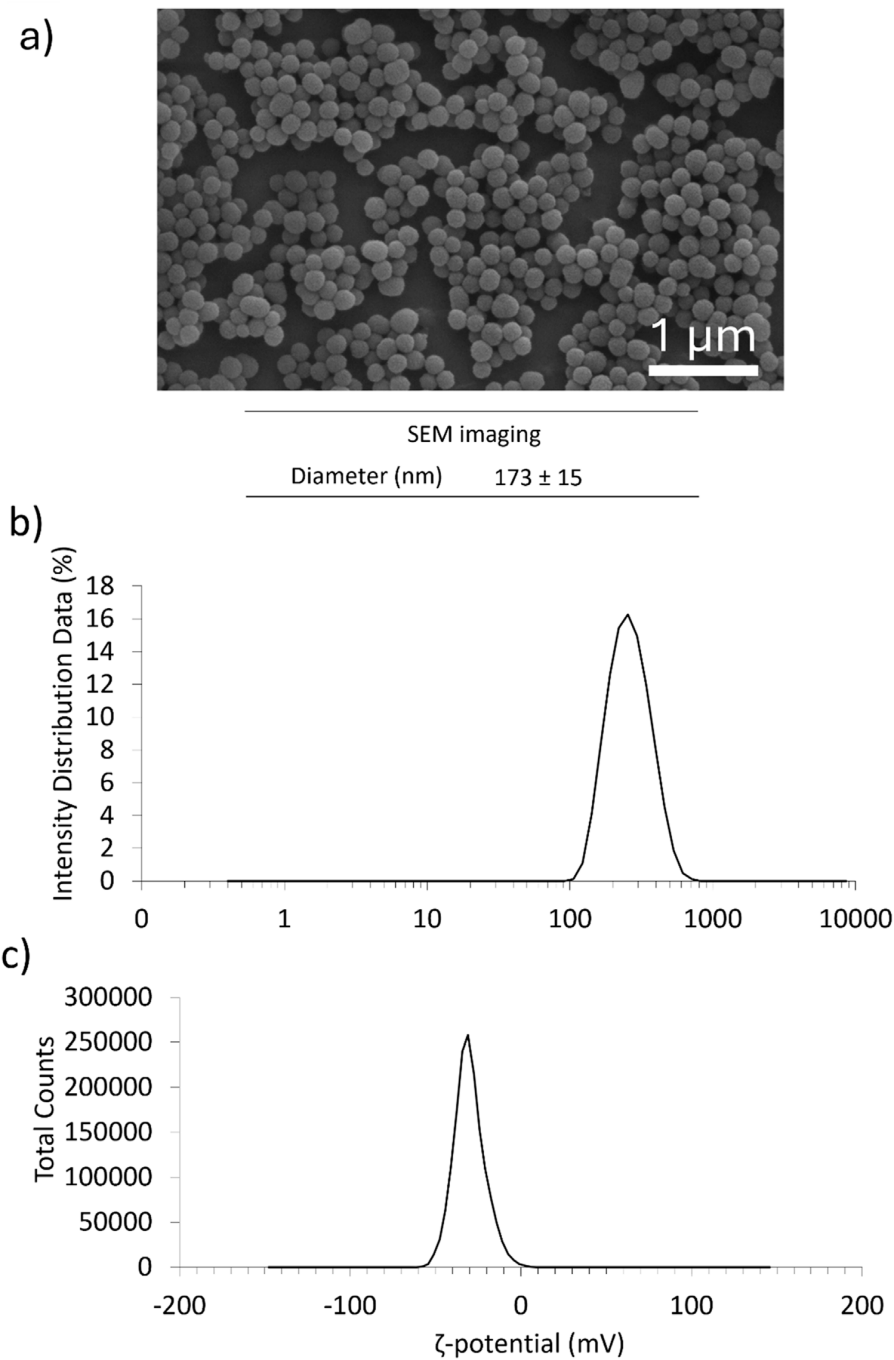
Characterization of PDNPs. **(a)** Representative SEM image of PDNPs and the average diameter of the nanostructures. **(b)** DLS analysis of PDNPs; **(c)** zeta potential analysis of PDNPs.

PDNPs were successfully synthesized via oxidative self-polymerization and exhibited a spherical morphology with uniform size distribution, as confirmed by SEM imaging (Figure 1a). The average diameter was approximately 173 ± 15 nm. Dynamic light scattering analysis revealed a hydrodynamic diameter of 210 ± 2 nm and a low polydispersity index (PDI = 0.04 ± 0.03), indicating a high degree of monodispersity (Table 1; Figure 1b). The ζ-potential was measured at −42.6 ± 0.3 mV, confirming strong surface charge and colloidal stability (Table 1; Figure 1c).

**TABLE 1:** DLS analysis of PDNPs.

DLS analysis	
Diameter (nm)	210 ± 2
PDI	0.04 ± 0.03
ζ-potential (mV)	−42.6 ± 0.3

### 3.2 Biocompatibility of PDNPs

**FIGURE 2:**
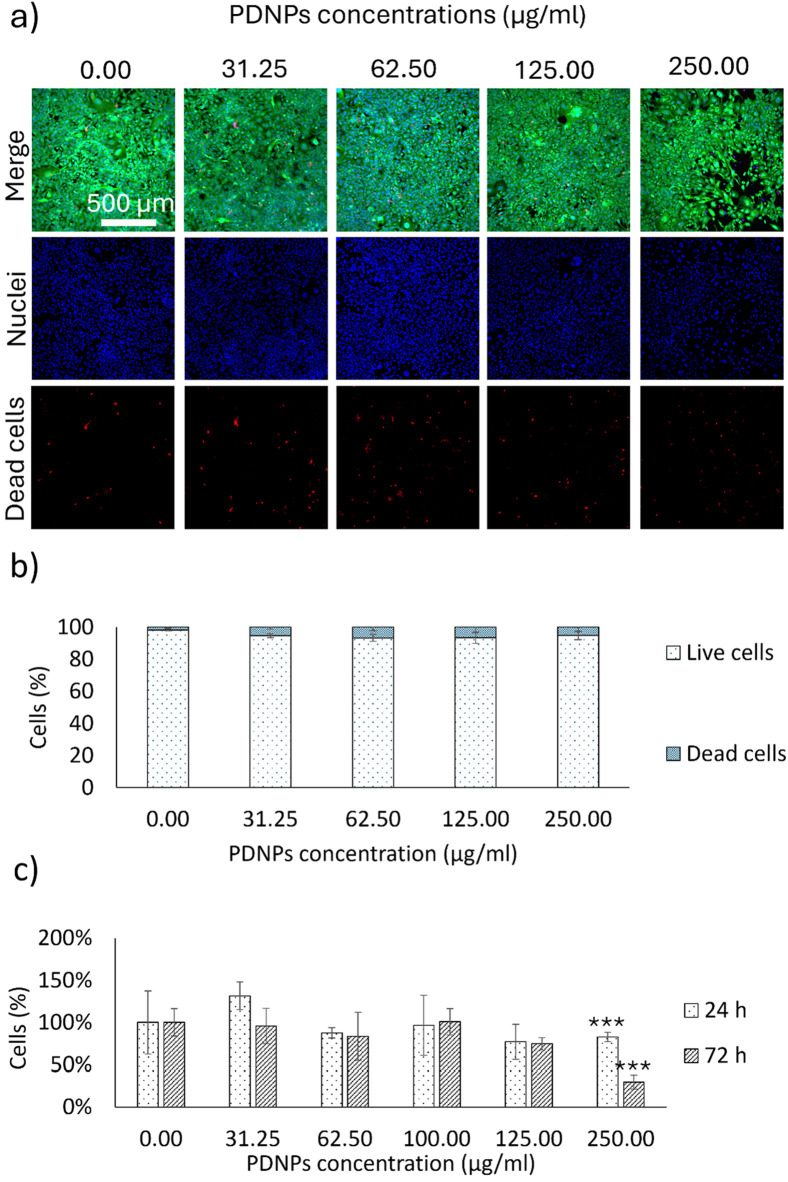
**(a)** Representative Live/Dead images of microglia (HMC3) cells treated with various concentrations of PDNPs and **(b)** corresponding quantitative analysis; **(c)** Picogreen® analysis of microglia (HMC3) cells treated with various concentrations of PDNPs (***p < 0.001).

Live/Dead assays demonstrated that PDNPs were well tolerated by HMC3 microglial cells across a wide range of concentrations (31.25–250 μg/mL) over 72 h. More than 90% of cells remained viable at all concentrations, with no significant difference from the untreated control (p > 0.05, Figures 2a,b). Quantification via PicoGreen™ assays showed that at concentrations above 125 μg/mL, there was a statistically significant reduction in DNA content, suggesting reduced cell proliferation or viability at higher doses (p < 0.001, Figure 2c). Based on these findings, 100 μg/mL was selected as the optimal concentration for subsequent experiments.

### 3.3 Cellular uptake and localization of PDNPs

**FIGURE 3:**
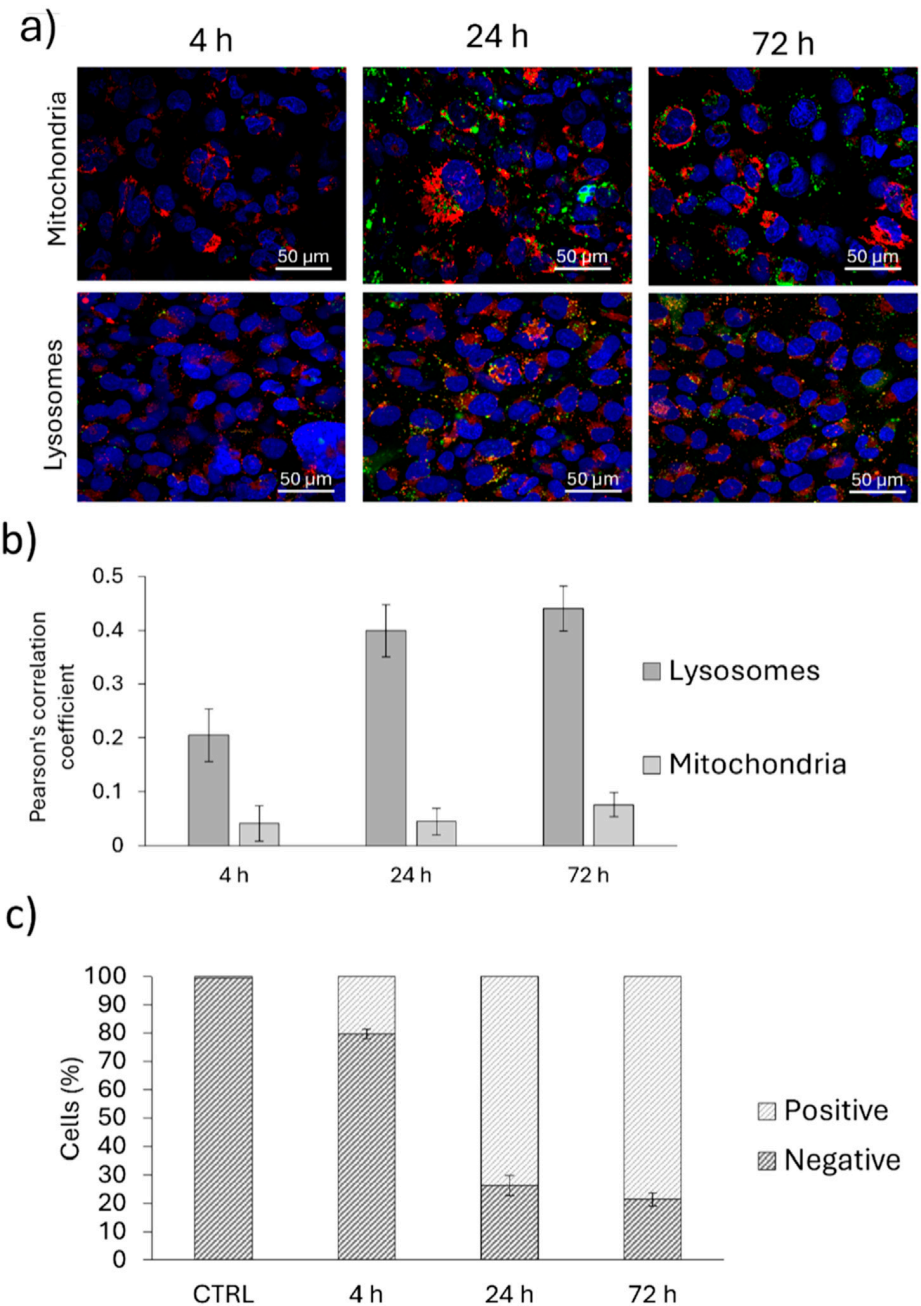
**(a)** Representative confocal images of microglia (HMC3) cells showing the intracellular localization of PDNPs with respect to mitochondria and lysosomes, and **(b)** corresponding Pearson’s correlation coefficient; **(c)** Flow cytometry analysis showing the internalization rate of PDNPs in HMC3 cells at various time points.

The internalization of PDNPs was assessed through confocal microscopy (Figures 3a,b) and flow cytometry (Figure 3c). Confocal microscopy confirmed nanoparticle accumulation primarily in lysosomes, with minimal localization in mitochondria. Pearson’s correlation coefficients for colocalization with lysosomes increased from 0.205 ± 0.049 at 4 h to 0.441 ± 0.041 at 72 h, while colocalization with mitochondria remained below 0.08 throughout, indicating targeted lysosomal accumulation. Flow cytometry revealed progressive internalization of DiO-labeled PDNPs over time, with increasing fluorescence detected at 4, 24, and 72 h post-exposure.

### 3.4 PDNPs reduce ROS in IFN-Stimulated microglia

**FIGURE 4:**
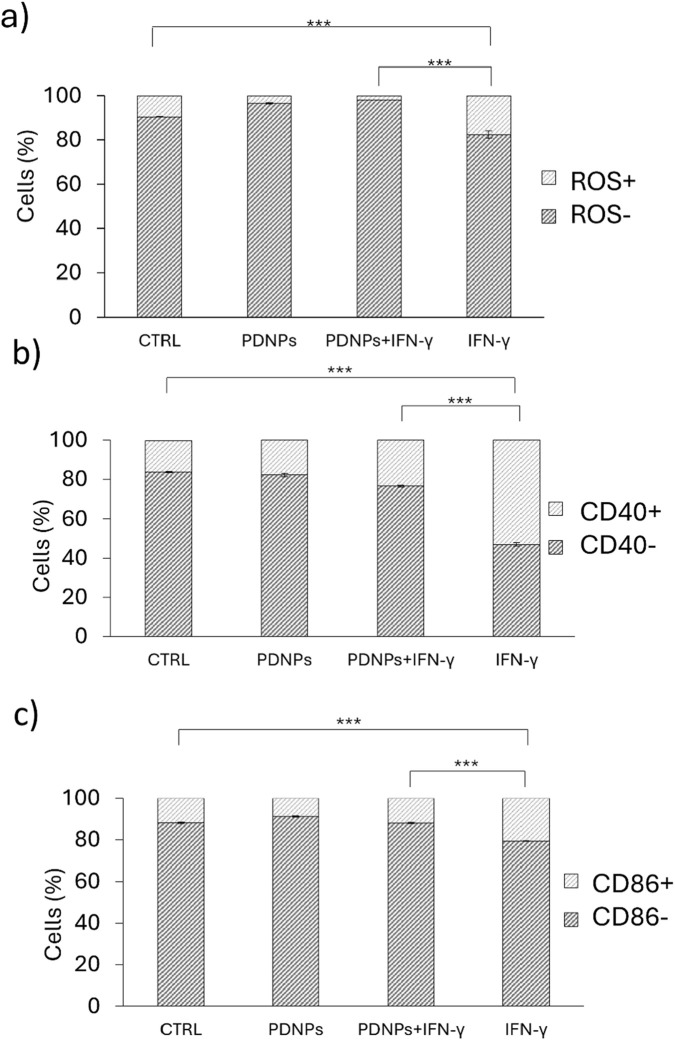
**(a)** Flow cytometry analysis showing the relative ROS levels of microglia (HMC3) in various experimental conditions; **(b)** Flow cytometry analysis showing the relative CD40 expression levels of microglia (HMC3) cells in various experimental conditions and **(c)** Flow cytometry analysis showing the relative CD86 expression levels of microglia (HMC3) cells in various experimental conditions.

PDNPs were able to significantly reduce the amount of ROS-positive cells in control cells (from 9.52% ± 0.14%–3.43% ± 0.33, p < 0.001, Figure 4a). Treatment with IFN-γ alone significantly increased intracellular ROS levels (17.57% ± 1.65 positive cells). In contrast, co-treatment with PDNPs and IFN-γ reduced ROS expression to 1.91% ± 0.09, a level comparable to that of untreated controls and significantly lower than that of the IFN-γ group (p < 0.001, Figure 4a). These findings confirm the strong antioxidant effect of PDNPs in microglial cells under inflammatory stress (representative flow cytometry histograms are shown in Supplementary Figure S1).

### 3.5 PDNPs suppress M1 marker expression

Flow cytometric analysis revealed that IFN-γ treatment resulted in a significant upregulation of the M1 surface markers CD40 and CD86. CD40-positive cells increased to 52.99% ± 0.90, compared to 16.03% ± 0.35 in controls. Co-treatment with PDNPs significantly reduced CD40 expression to 23.44% ± 0.39 (p < 0.001, Figure 4b). Similarly, CD86 expression increased to 20.68% ± 0.22 with IFN-γ alone compared to 11.83% ± 0.38 in the control, while PDNPs + IFN-γ reduced it to 11.99% ± 0.31, close to control levels (p < 0.001, Figure 4c). These results demonstrate that PDNPs effectively prevent the phenotypic switch toward the pro-inflammatory M1 state (Representative flow cytometry histograms are shown in Supplementary Figures S2, S3.

### 3.6 PDNPs decrease pro-inflammatory cytokine production

**FIGURE 5:**
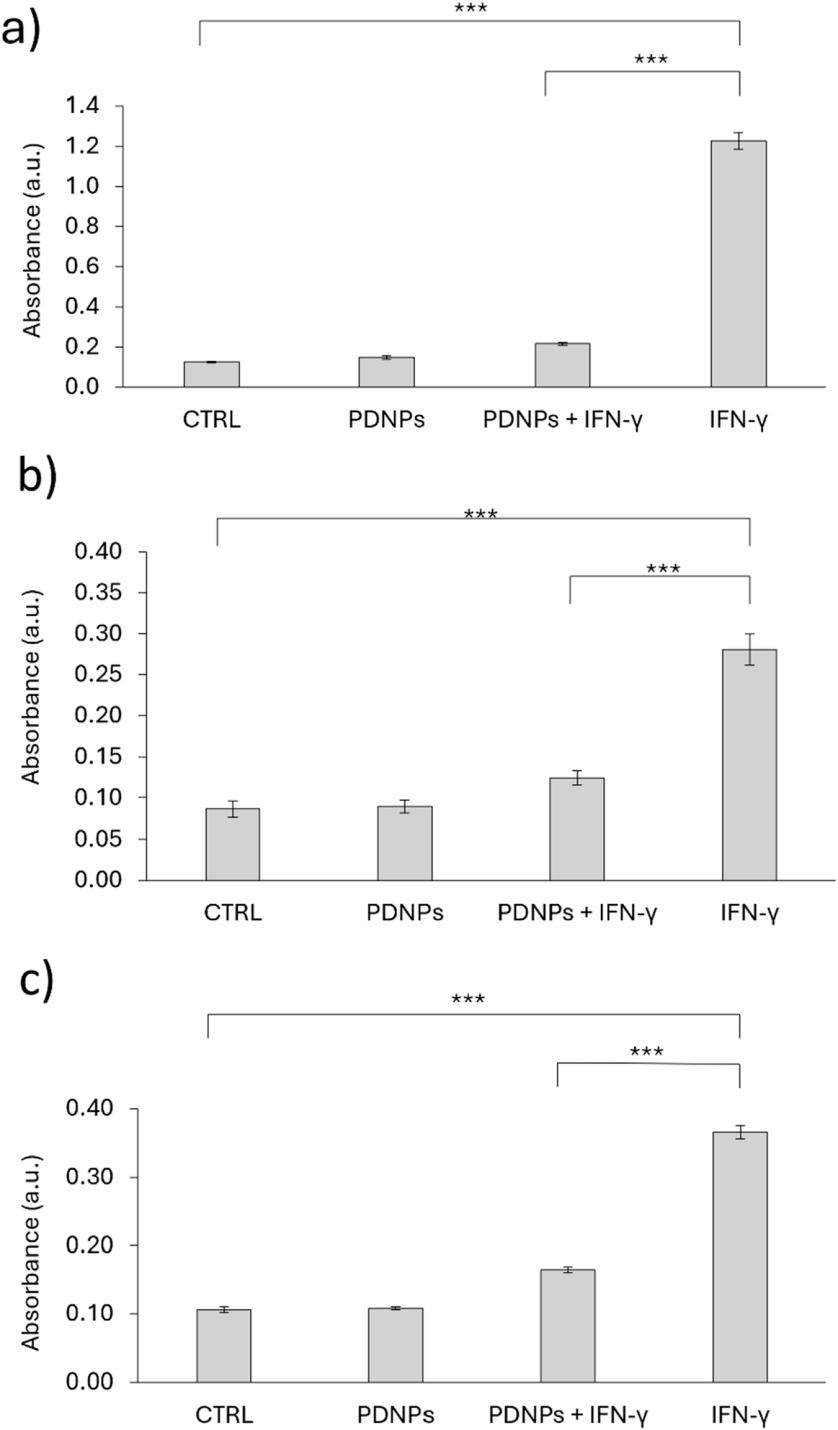
ELISA analysis showing the relative production of **(a)** IL-6, **(b)** IL-8, and **(c)** TNF-α of microglia (HMC3) cells in various experimental conditions.

ELISA assays revealed that IFN-γ stimulation significantly increased secretion of IL-6, IL-8, and TNF-α. PDNP co-treatment attenuated this response: IL-6 (Figure 5a): From 1.226 ± 0.040 (IFN-γ) to 0.215 ± 0.009 (PDNPs + IFN-γ); IL-8 (Figure 5b): From 0.281 ± 0.019 (IFN-γ) to 0.124 ± 0.009 (PDNPs + IFN-γ); TNF-α (Figure 5c): From 0.366 ± 0.010 (IFN-γ) to 0.165 ± 0.004 (PDNPs + IFN-γ); All reductions were statistically significant (p < 0.001), confirming that PDNPs suppress inflammatory cytokine release in activated microglia.

## 4 Discussion

As mentioned in the introduction, neuroinflammation is a well-established contributor to the progression of several CNS disorders, including AD, PD, and ALS. Among the cellular drivers of this process, microglia (resident immune cells of the CNS) play a pivotal role. (C et al., 2005; Bartels et al., 2020; Clarke and Patani, 2020). Although their physiological function includes surveillance, debris clearance, and support of neuronal health, their dysregulated activation, particularly toward the pro-inflammatory M1 phenotype, contributes to a harmful cycle of oxidative stress, cytokine release, and neuronal damage. (Guo et al., 2022; Tang and Le, 2016; Honjoh et al., 2019). The therapeutic modulation of microglial phenotype has thus emerged as a compelling strategy to interrupt this cycle and protect neural tissues. The present study demonstrates that PDNPs (fully organic, melanin-like nanomaterials with intrinsic antioxidant properties) can effectively attenuate IFN-induced M1 microglial activation. Our findings provide novel evidence supporting the use of PDNPs as immunomodulatory agents that can mitigate inflammation and oxidative stress in microglia, thereby opening new perspectives for their application in nanomedicine-based strategies for treating neurodegenerative diseases. The PDNPs synthesized in this study displayed well-controlled morphology and surface characteristics, with an average hydrodynamic diameter of 210 nm and a strongly negative surface ζ-potential (−43 mV). These properties are consistent with prior literature and support stable dispersion and efficient cellular internalization. (Battaglini et al., 2020; Battaglini et al., 2022). Notably, SEM and DLS data confirmed a monodisperse population, indicating reproducibility and suitability for biological applications. Our biocompatibility assays demonstrated that PDNPs are well tolerated by HMC3 cells over a range of concentrations. While high doses (≥125 μg/mL) slightly reduced DNA content, suggesting some impact on proliferation, no significant cytotoxicity was observed even at 250 μg/mL. This result aligns with earlier reports describing PDA’s minimal cytotoxicity and reinforces the material’s promise for CNS applications, where safety and biodegradability are crucial. (Battaglini et al., 2024b). Fluorescence-based uptake assays and confocal imaging confirmed that PDNPs are efficiently internalized by HMC3 cells and predominantly accumulate within lysosomes. Their minimal mitochondrial localization suggests a limited risk of mitochondrial stress or interference with ATP synthesis, a notable advantage over some inorganic nanoparticles. (Tee et al., 2016; De Matteis, 2017; Xiang et al., 2022). The lysosomal targeting may also play a functional role in modulating immune signaling, as lysosomes are known to be key organelles for inflammation-related pathways such as inflammasome activation and cytokine processing. (Tee et al., 2016; Quick et al., 2023). One of the key outcomes of this study is the demonstration that PDNPs significantly reduce IFN-induced ROS production. IFN-γ is a potent activator of M1 microglial polarization, triggering oxidative stress through enhanced mitochondrial respiration and NADPH oxidase activity. (Moran et al., 2007). PDNPs likely neutralize ROS through redox cycling of their surface catechol and quinone groups, acting as electron donors that stabilize radical species. (Guo et al., 2021). This mechanism is supported by prior studies showing PDA’s efficacy in scavenging superoxide anions (Deng et al., 2024) and hydroxyl radicals (Lei et al., 2025). In tandem with their antioxidant activity, PDNPs also suppressed the expression of known M1 surface markers (CD40 and CD86) following IFN-γ stimulation. (Ebner et al., 2013).These markers are associated with antigen presentation and co-stimulation of T cells, and their upregulation is indicative of a pro-inflammatory immune response. The reduction in CD40/CD86 expression is consistent with an attenuation of M1 activation and may involve effects on transcriptional or signaling pathways, potentially mediated by the observed decrease in oxidative stress. The. Complementing these phenotypic changes, we observed substantial reductions in the secretion of pro-inflammatory cytokines (TNF-α, IL-6, and IL-8) in PDNP-treated cells. These cytokines are central mediators of neuroinflammation and are elevated in multiple neurodegenerative disorders. (Smith et al., 2012; Ogunmokun et al., 2021; Zhang et al., 2023). TNF-α is known to exacerbate synaptic dysfunction and neuronal apoptosis, while IL-6 and IL-8 contribute to blood–brain barrier disruption and the recruitment of immune cells. (Versele et al., 2022). By attenuating the release of these cytokines, PDNPs may help restore a more homeostatic microenvironment in the brain. While the precise molecular mechanisms remain to be elucidated, it is plausible that PDNPs interfere with IFN-γ downstream signaling, possibly affecting transcription factors such as NF-κB or STAT1. (Hu and Ivashkiv, 2009). Alternatively, the observed effects could arise indirectly from the reduction in intracellular ROS, which are known secondary messengers in inflammatory signaling cascades. (Fan et al., 2023). Additionally, the lysosomal localization of PDNPs may disrupt endosomal maturation or modulate the presentation of inflammatory ligands, contributing to phenotype modulation.

## 5 Conclusion

This study demonstrates that PDNPs possess significant immunomodulatory potential in the context of neuroinflammation. PDNPs were shown to be biocompatible, effectively internalized by human microglial cells, and capable of attenuating key features of M1 microglial activation induced by IFN-γ. Specifically, PDNP treatment reduced intracellular ROS, downregulated M1 surface markers CD40 and CD86, and suppressed the secretion of pro-inflammatory cytokines TNF-α, IL-6, and IL-8. These results position PDNPs as promising nanotherapeutic agents capable of modulating microglial behavior in a controlled, non-toxic manner. By reducing oxidative stress and attenuating pro-inflammatory markers, PDNPs show potential to modulate microglial activation in a manner that may be relevant to neurodegenerative diseases characterized by chronic neuroinflammation. Their organic, metal-free composition further enhances their safety profile compared to many other nanoparticle systems, making them suitable candidates for therapeutic development within the central nervous system. Looking forward, several key steps are necessary to further validate and expand upon these findings. First, additional studies in more physiologically relevant models (such as 3D microglial spheroids, neuron-glia co-cultures, and *in vivo* animal models) will be critical to assess the translational potential of PDNPs. Evaluating their pharmacokinetics, ability to cross the blood–brain barrier, and long-term effects in diseased brain tissue will be essential for clinical progression. A limitation of this study is the use of an immortalized microglial cell line (HMC3), which may not fully replicate the metabolic and inflammatory responses of primary microglia; therefore, future studies will be required to confirm these findings in primary cultures and *in vivo* models to strengthen their translational relevance. Furthermore, investigating the impact of PDNPs on the anti-inflammatory M2 microglial phenotype could provide insight into whether they merely suppress inflammation or also promote repair. Exploring interactions with other key inflammatory stimuli (such as lipopolysaccharide, amyloid-β, and α-synuclein) will also help determine if PDNP could be exploited to treat different CNS diseases. Finally, the surface chemistry of PDNPs allows for a wide range of functionalization strategies. Future work may explore the conjugation of targeting ligands, drugs, or imaging agents to create multifunctional, disease-specific delivery systems capable of crossing biological barriers and acting with high spatial precision. In summary, PDNPs represent a versatile and promising tool in the fight against neuroinflammatory conditions. With further development and validation, they may serve as a foundation for novel therapeutic approaches targeting the immune dysfunctions involved in neurodegeneration.

## Data Availability

The original contributions presented in the study are included in the article/Supplementary Material, further inquiries can be directed to the corresponding authors.
