# Supplementary material for: Polydopamine nanoparticles as immunomodulators: inhibition of M1 microglial polarization
Source: Front Bioeng Biotechnol. 2025 Oct 17;13:1672520. doi: 10.3389/fbioe.2025.1672520 (PMC12575223; doi:10.3389/fbioe.2025.1672520)
Supplement: Supplementary file 1 [file DataSheet1.docx]

*Supplementary Information*

**Polydopamine Nanoparticles as Immunomodulators: Inhibition of M1 Microglial Polarization**

Maria Cristina Ceccarelli*^1,2^, Luigi Lai*^1,3^, Alessio Carmignani^1^, Matteo Battaglini*^1^,Gianni Ciofani*^1^

^1^ Istituto Italiano di Tecnologia, Smart Bio-Interfaces, Viale Rinaldo Piaggio 34, Pontedera, 56025, Italy.

^2^ Scuola Superiore Sant’Anna, the Biorobotics Institute, Viale Rinaldo Piaggio 34, Pontedera, 56025, Italy

^3^ Politecnico di Torino, Department of Mechanical & Aerospace Engineering, Corso Duca degli Abruzzi 24, Torino, 10129, Italy.


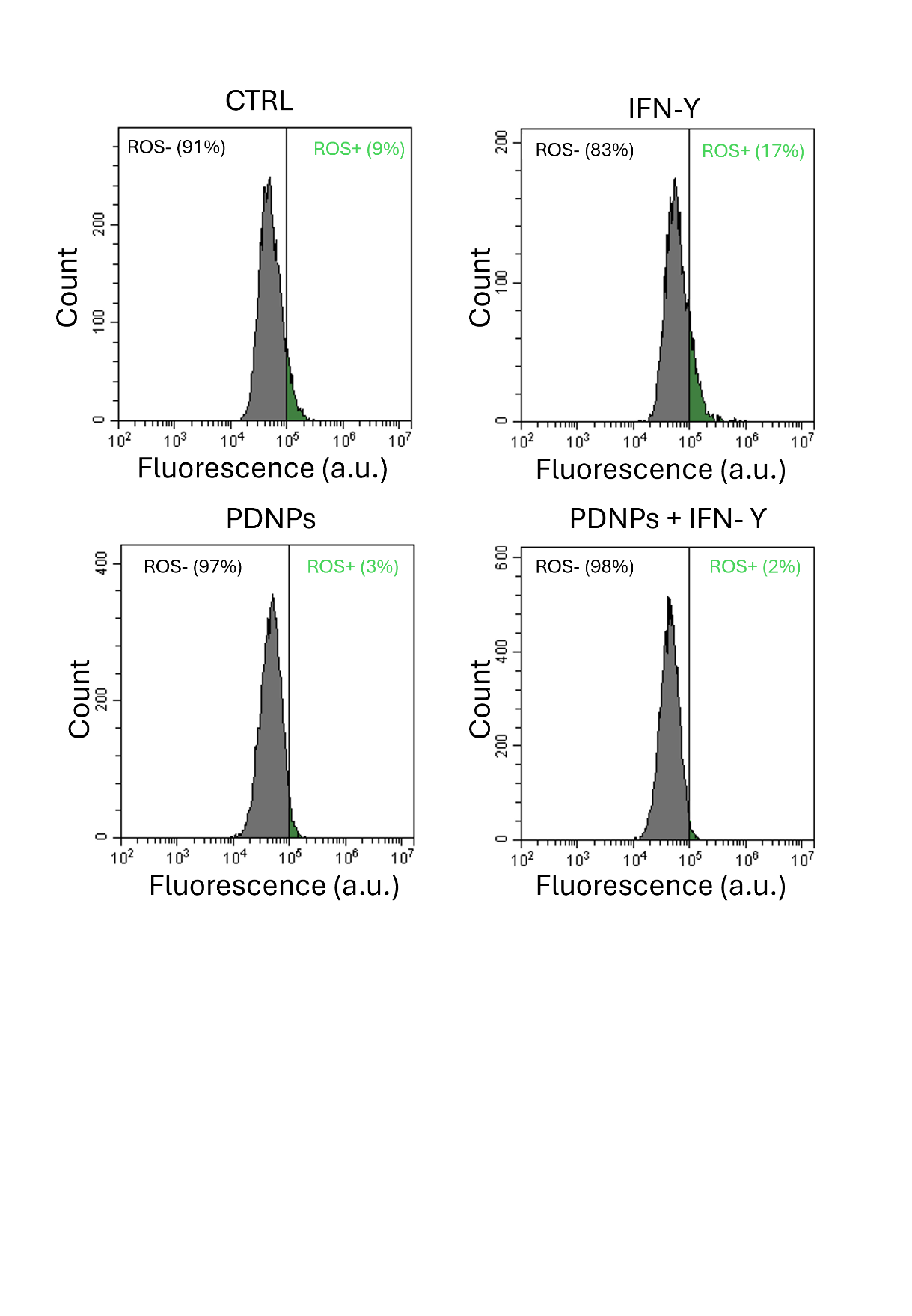


**Figure S1:** Representative flow cytometry histograms showing the ROS levels in HMC3 cells in various experimental conditions.


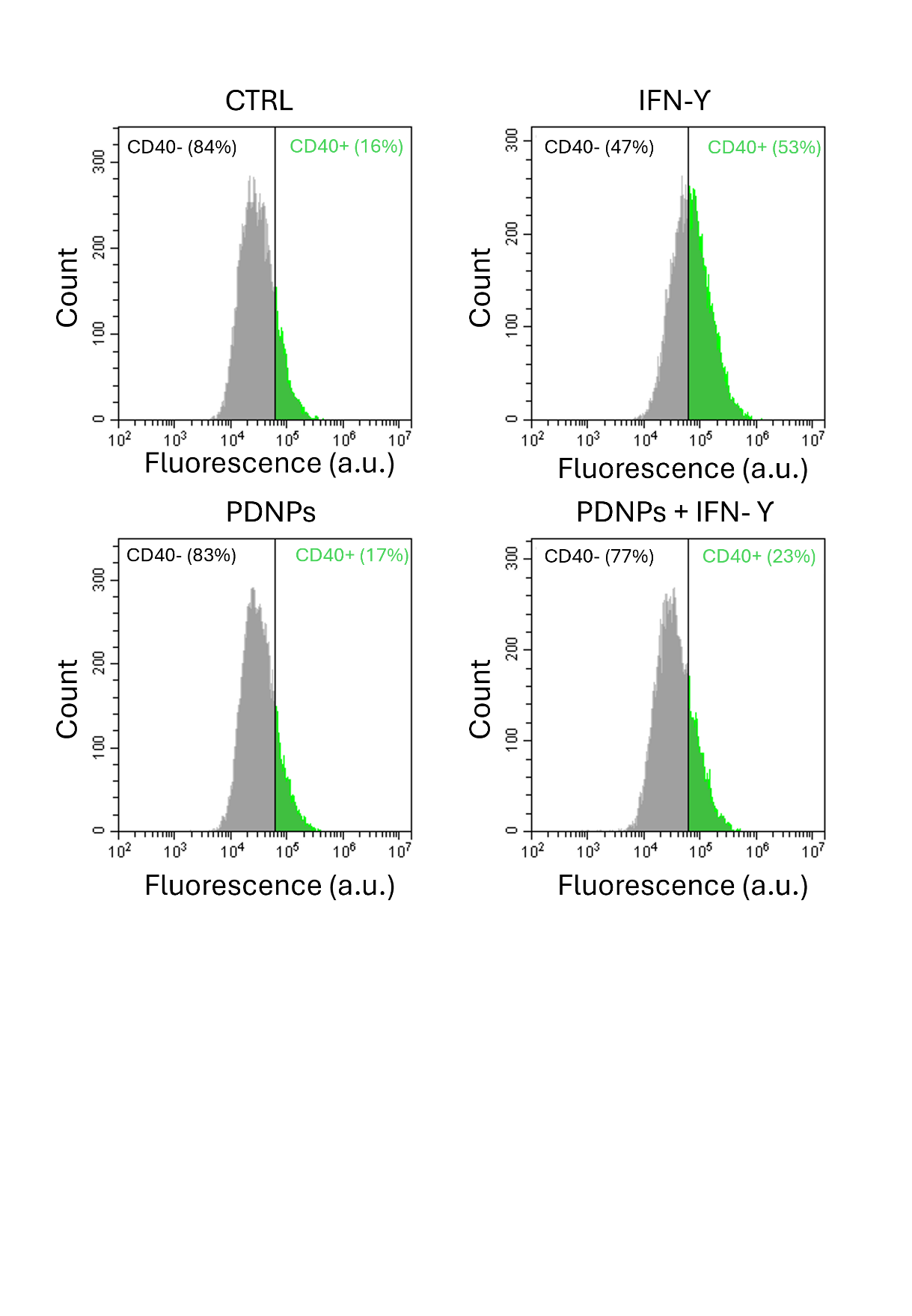


**Figure S2:** Representative flow cytometry histograms showing the CD40 expression levels in HMC3 cells in various experimental conditions.


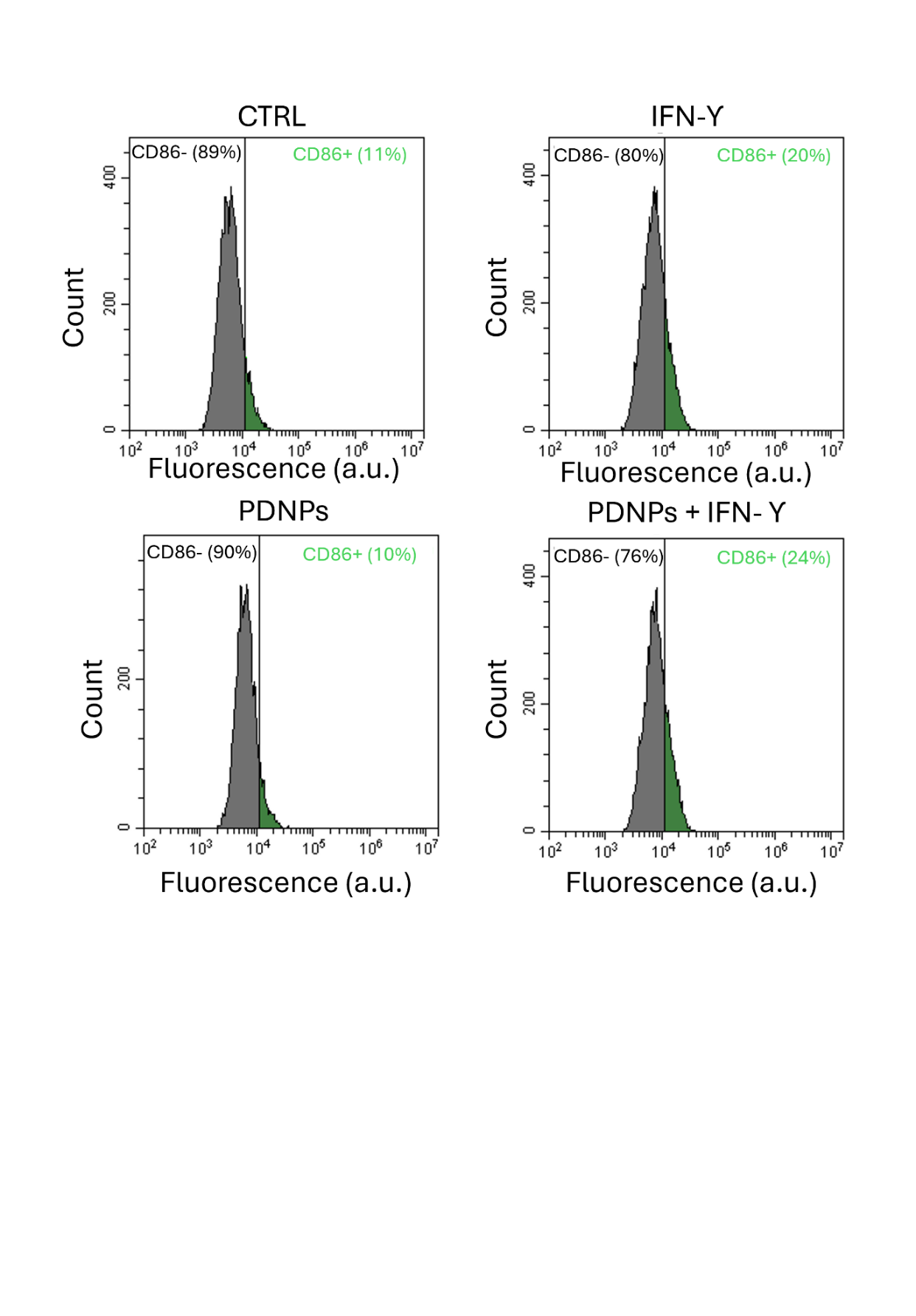


**Figure S3:** Representative flow cytometry histograms showing the CD86 expression levels in HMC3 cells in various experimental conditions.
